# Exploring Varied Treatment Strategies for Metabolic Dysfunction-Associated Steatotic Liver Disease (MASLD)

**DOI:** 10.3390/life14070844

**Published:** 2024-07-03

**Authors:** Amani Elshaer, David M. H. Chascsa, Blanca C. Lizaola-Mayo

**Affiliations:** 1 Department of Internal Medicine, Mayo Clinic, Scottsdale, AZ 85054, USA; 2 Division of Gastroenterology and Hepatology, Mayo Clinic, Scottsdale, AZ 85054, USA; 3 Transplant Center, Department of Medicine, Mayo Clinic, Scottsdale, AZ 85054, USA

**Keywords:** metabolic-associated steatotic liver disease, fibrosis, cirrhosis, obesity, weight loss, lifestyle modification, pharmacotherapy, bariatric surgery

## Abstract

Metabolic dysfunction-associated steatotic liver disease (MASLD) represents a liver disorder characterized by steatosis with underlying metabolic risk factors. The prevalence of MASLD continues to rise, leading to increased patient risk of various complications. Recent research has been focused on new therapeutic strategies to reduce the incidence of MASLD and provide effective treatment plans to prevent further irreversible liver damage. The treatment approach is multifactorial, with a primary focus on weight loss and management of underlying comorbidities through lifestyle modifications, pharmacotherapy, or surgical options. Ongoing research is exploring new pharmacological therapies that could enhance the treatment of MASLD.

## 1. Introduction

**Figure 1:**
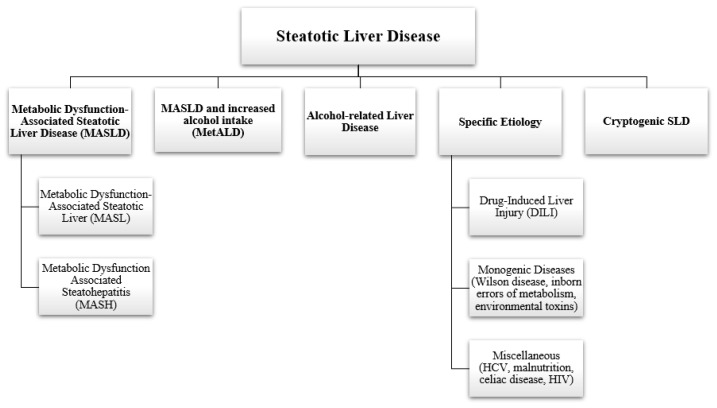
Steatotic liver disease and its subcategories. Steatotic liver disease encompasses various subcategories, including metabolic dysfunction-associated steatotic liver disease (MASLD); the overlapping condition of MASLD plus significant alcohol consumption or alcohol use disorder (MetALD); alcohol-related liver disease; cases with specific identifiable etiology; and lastly, diseases with a non-identifiable etiology (cryptogenic). Within MASLD, further distinctions are made: MASLD denotes the presence of hepatic steatosis without steatohepatitis, while MASH indicates the coexistence of steatohepatitis [1].

**Figure 2:**
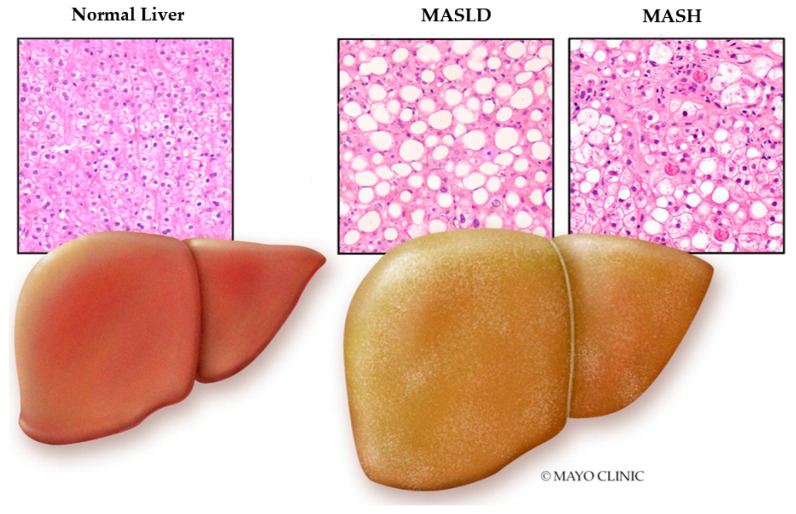
Normal liver tissue on histology is characterized by uniformly arranged hepatocytes, which are separated by sinusoids with minimal fat accumulation. In contrast, MASLD is distinguished by the accumulation of fat droplets. Over time, this can progress to inflammation, which is marked by the presence of inflammatory cell infiltrates, including neutrophils, which can lead to MASH.

**Figure 3:**
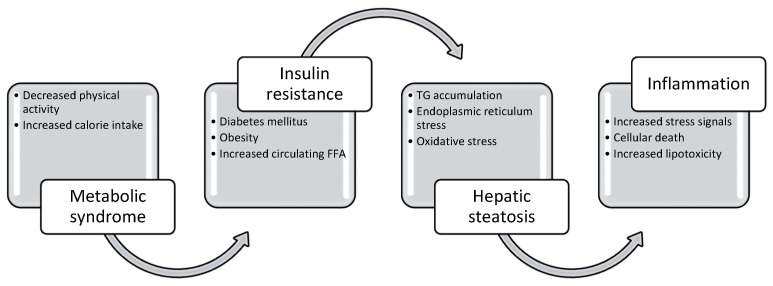
Pathogenesis of MASLD. The imbalance between low physical activity and increased calorie intake will result in the development of metabolic syndrome and increased insulin resistance. Insulin resistance increases the levels of free fatty acids (FFAs) in hepatocytes, leading to more inflammation and triglyceride (TG) accumulation. The ongoing stressful environment within the hepatocyte cells then causes inflammation and cellular death.

Metabolic dysfunction-associated steatotic liver disease (MASLD) is the updated nomenclature for what was previously known as nonalcoholic fatty liver disease (NAFLD) and falls under the umbrella of steatotic liver disease, as illustrated in Figure 1 [1]. The updated terminology reflects the importance of metabolic dysfunction in the pathophysiology of the disease [1]. MASLD is defined by the presence of ≥5% hepatic steatosis with at least one metabolic risk factor (overweight, hyperglycemia, hypertension, hyperlipidemia) in the absence of other causes of steatosis, such as medications, alcohol use, viral hepatitis, or other illnesses [2]. MASLD is a spectrum of liver conditions that may range from simple hepatic steatosis to metabolic dysfunction-associated steatohepatitis (MASH) when there is histologic evidence of inflammation and hepatocellular injury (hepatocyte ballooning with or without fibrosis) (Figure 2) [2]. With continued injury, it may cause progressive liver fibrosis, and ultimately cirrhosis (Figure 3) [2].

Apart from liver-related complications, MASLD is linked to increased cardiovascular complications, such as myocardial infarction, ischemic stroke, heart failure, and cardiovascular death [3,4]. Cardiovascular complications are the leading cause of mortality in patients with MASLD [4]. Additionally, MASLD is associated with increased metabolic complications, such as diabetes mellitus, kidney disease, sarcopenia, and cancer [5,6,7,8,9].

The management of MASLD is focused on effectively addressing the underlying risk factors for the development of the disease and preventing further disease progression. Early identification and treatment initiation can potentially reduce the risk of complications and the associated morbidity and mortality [10].

In this article, we extensively review the variety of guideline-based treatment modalities for patients with MASLD, beginning with lifestyle modifications and extending to medications and surgical procedures. Additionally, we discuss recently approved medical treatments, meticulously detailing the associated side effects and elucidating the clinical implications for individuals with MASLD.

## 2. Epidemiology

MASLD is considered the most common liver disease, affecting 30% of the population worldwide [11]. In the USA, the prevalence of MASLD has increased from 30.8% in 1999–2000 to 37.7% in 2017–2018, mirroring the global rise in obesity, hypertension, hyperlipidemia, type 2 diabetes, and metabolic syndrome [11]. The risk of progressing to MASH is present in 20–30% of MASLD cases, which is also linked to liver-related and cardiovascular complications. In a large cohort study, it was observed that 7–10% of patients diagnosed with MASH progress to have cirrhosis over a period of 10 years [12]. The increased trend in MASLD reflects the importance of early screening and prevention. Additionally, identifying high-risk populations and providing adequate treatment will reduce the overall burden and clinical implications associated with MASLD [7].

## 3. Pathophysiology

To understand the management of MASLD, it is crucial to understand the underlying pathogenesis to develop targeted therapeutic interventions. The pathogenesis of MASLD is a multifactorial complex interplay that involves metabolic dysfunction, genetic predisposition, and environmental risk factors [13].

MASLD is driven by imbalances due energy intake that exceeds the metabolic needs of the body, and thus, results in the accumulation of lipids within hepatocytes referred to as hepatic steatosis (the central feature of MASLD). Studies showed that dietary factors influence the development of MASLD. Diets high in fat and fructose lead to increased insulin resistance, inflammation, and fibrosis by increasing the expression of genes for liver fibrosis and inflammation [14].

One of the key drivers of hepatic steatosis is insulin resistance. The function of insulin normally promotes glucose uptake and storage in peripheral tissues and inhibits lipolysis in adipose tissue [15]. In states where there is increased insulin resistance, adipose tissue will continuously release fatty acids, leading to increased fatty acid deposition in the liver [15]. Additionally, insulin resistance will increase de novo lipogenesis within the liver by converting extra carbohydrates into fatty acids [16]. The increased lipid accumulation in the hepatocytes will eventually lead to lipotoxicity by inducing cellular stress and damage within the hepatocytes [17]. The mitochondria becomes dysfunctional and impairs fatty acid oxidation, leading to the retention of triglycerides within hepatocytes. The accumulated lipids will induce the cellular stress response by increasing the endoplasmic reticulum stress and oxidative stress. This will then result in lipid peroxidation, which contributes to further cellular damage and inflammation. As a result, hepatic steatosis may progress to MASH in the setting of increased inflammation and liver cell injury. The pathogenesis of MASLD is summarized in Figure 3.

**Figure 4:**
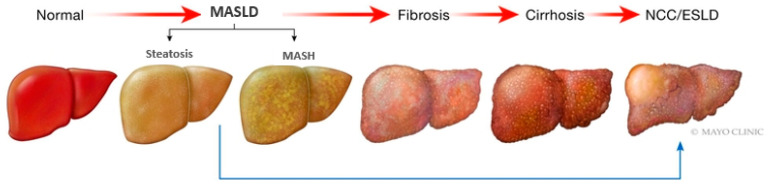
MASLD disease progression. This visual representation illustrates the trajectory of liver disease evolving into MASLD (steatosis). As the liver sustains ongoing damage, MASLD advances from initial stages to fibrosis, then culminates in cirrhosis (extensive fibrosis with regenerative nodules), and ultimately progresses to end-stage liver disease (ESLD)/decompensated liver disease.

The activation of the inflammatory pathways will contribute to the recruitment of immune cells to the liver. Continued inflammation and hepatocytes will initiate the activation of the wound-healing response. This process activates hepatic stellate cells, leading to the subsequent synthesis of matrix proteins, like collagen. The cumulative effect is the development of fibrosis, which holds the potential to progress to cirrhosis (Figure 4). Cirrhosis is defined by the distortion of the liver architecture due to extensive scarring, leading to impaired liver function [18]. Therefore, therapies are currently targeting these risk factors, including regulating insulin resistance, lowering lipid levels, and promoting weight loss. Ongoing research is also investigating other therapeutic targets, such as FGF21 analogs, lipogenesis inhibitors, and microbiome modulation; this aims to address the multifactorial pathogenesis of MASLD [19].

## 4. Diagnosis

There should be high clinical suspicion for MASLD in patients that present with metabolic syndrome, with or without elevated liver enzymes, and thus, warrant further evaluation. Several non-invasive diagnostic methods are available to evaluate for MASLD, including ultrasound, vibration-controlled transient elastography (VCTE), magnetic resonance elastography, and non-invasive fibrosis tests (FIB-4 score). However, the gold standard diagnostic method is a liver biopsy. A liver biopsy helps to quantify the level of steatosis, hepatocyte ballooning, and necro-inflammatory score (Figure 3). These factors are then summed into the NAFLD activity score (NAS) (Table 1), which includes steatosis (0–3 points), ballooning (0–2 points), and inflammation (0–3 points) [20]. In patients diagnosed with MASLD or MASH, it is important to determine the stage and degree of fibrosis, as it will influence the prognosis. Stage 0 indicates no fibrosis; stage 1 is pericellular fibrosis; stage 2 is pericellular and portal fibrosis; stage 3 is bridging fibrosis; and stage 4 is cirrhosis [21].

## 5. Lifestyle Modifications

### 5.1. Weight Loss

Lifestyle management, including diet and physical activity, are the foundations of MASLD management. Weight reduction was shown to improve liver histology and metabolic parameters in numerous studies. A randomized controlled trial by the American Gastroenterology Association (AGA) demonstrated notable improvements in steatosis among patients who achieved a 5% reduction in their total body weight (TBW). Furthermore, among those who achieved a 7% reduction in their TBW, 64% had MASH resolution. In patients who lost 10% of their TBW, 45% had fibrosis regression by at least one stage, whereas the remaining 55% had fibrosis stabilization [22]. In another prospective study by Vilar-Gomez et al., 293 patients with histologically proven MASH were encouraged to implement lifestyle changes for weight reduction over 52 weeks. Liver biopsies were obtained before and after the intervention. The results show the resolution of steatohepatitis in 25% of the patients, 19% experienced regression of fibrosis, and 47% had reductions in their nonalcoholic fatty liver disease score (NAS). Patients with higher weight loss (≥10%) had a 90% resolution of NASH [23]. A meta-analysis including 2809 patients showed a 0.03 point reduction (*p* < 0.0001) in steatosis by histology or ultrasound for every 1 kg weight loss, with improvements in both AST and ALT [24]. These findings demonstrate the significant impact of weight loss on the different stages of MASLD. Therefore, a gradual weight loss of 5–10% of TBW is recommended by the American Association for the Study of Liver Diseases (AASLD) and AGA for the management of MASLD [22].

### 5.2. Dietary Recommendations

The Mediterranean diet has been extensively researched for patients diagnosed with MASLD/MASH. This dietary approach includes a rich variety of vegetables, fruits, whole grains, and healthy fats while minimizing the consumption of dairy, red processed meat, and carbohydrates (especially sugars and refined carbohydrates). Instead, it encourages the consumption of monounsaturated fatty acids (MUFAs), saturated fatty acids (SFAs), and omega-3 fatty acids, which were shown to lower LDL cholesterol and triglycerides (TG) while improving postprandial glucose levels [25]. These beneficial fats are mainly provided through sources such as olives; nuts; avocados; and certain fish, like mackerel and sardines. A meta-analysis found that implementing a Mediterranean diet reduces the risk of developing MASLD by 23% [26]. Not only does it reduce the risk for MASLD, but it can also produce reductions in cardiovascular risk, diabetes mellitus, and overall mortality [27]. This is due to the balance of the macronutrient composition and the richness of its micronutrients, making it the recommended dietary intervention for patients with MASLD [2]. Additionally, the Mediterranean diet provides around 11.7 mg/day of vitamin E, mainly through olive oil consumption [28]. As discussed below, vitamin E is shown to have multiple beneficial effects on MASLD due to its antioxidant properties. The European Association for the Study of the Liver (EASL), European Association for the Study of Diabetes (EASD), and the European Association for the study of Obesity (EASO) Practice Guidelines recommend implementing the Mediterranean diet for MASLD treatment, as it improves metabolism by reducing insulin resistance and lowering LDL levels and steatosis [29].

The Dietary Approaches to Stop Hypertension (DASH) diet was studied to determine its benefit on MASLD. It is a dietary approach designed to improve hypertension by emphasizing the consumption of fruits, vegetables, whole grains, and low-fat dairy products while reducing saturated fats, cholesterol, and refined grains. It is rich in potassium, magnesium, calcium, and protein. A RCT by Zade et al. involved 60 patients with MASLD [30]. Patients were randomized to consume a DASH diet versus a regular diet. Both diets were designed to be low calorie and include 52–55% carbohydrates, 16–18% protein, and 30% total fat for a total duration of 8 weeks. The results show that adherence to a DASH eating pattern helped to reduce alanine transaminase (ALT); alkaline phosphatase; triglycerides; the basal metabolic index (BMI); and inflammatory markers, such as C-reactive protein; as well as improve insulin metabolism, compared with the control group [30]. This beneficial effect was due to the DASH diet macronutrient content, including low fat levels.

An alternative dietary approach is to implement a calorie-restricted diet to achieve clinically significant weight loss. This was shown to improve insulin resistance, reduce intrahepatic fat, and normalize hepatic enzymes in patients with MASLD [31,32]. This approach targets a daily intake of 1200–1500 kcal or a reduction of 500–1000 kcal per day. Very low hypocaloric diets (800 kcal per day or less) were assessed in a small study involving women with obesity. It demonstrated the reversal of MASLD in those patients; however, the long-term effects of very low hypocaloric diets have not been explored [33].

Various other dietary approaches, such as vegan, low-carbohydrate, and low-fat diets [34]; early time-restricted feeding [35]; and intermittent fasting [36], were investigated in the management of MASLD. However, these trials were limited by the lack of liver biopsies and the heterogeneity of the study cohorts.

In conclusion, dietary recommendations should encourage implementing a diet rich in macronutrients with limited carbohydrates and saturated fats, such as the Mediterranean diet [2]. The dietary plan should focus on achieving weight loss through a sustainable long-term dietary plan. This holistic approach ensures that the dietary intervention is tailored to the individual preference with lasting health benefits [2].

### 5.3. Alcohol Consumption

Alcohol consumption significantly influences the progression of MASLD. According to the AASLD guidelines, alcohol intake is categorized as mild (20 g/day of alcohol in women and 30 g/day of alcohol in men), moderate (21–39 g/day in women and 31–59 g/day in men), and heavy (≥40 g/day in women and ≥60 g/day in men). Studies demonstrated that alcohol consumption not only accelerates liver injury but also interacts synergistically with other comorbidities, such as obesity and increased insulin resistance. A cross-sectional study by Blomdahl et al. revealed a correlation between moderate alcohol consumption among patients with type 2 diabetes and a significantly higher rate of fibrosis compared with those with minimal alcohol intake (50.0–60.0% vs. 3.3–21.6%, *p* < 0.05) [37].

Several mechanisms may explain the effect of alcohol on liver disease. Ethanol is metabolized by alcohol dehydrogenase and cytochrome P450 2EI (CYP2EI) in the liver, leading to the production of toxic acetaldehyde and an increased NADH/NAD+ ratio. This metabolic imbalance stimulates fatty acid synthesis while inhibiting their oxidation, ultimately resulting in steatosis. Additionally, the activity of CYP2EI generates reactive oxygen species, exacerbating oxidative stress, cellular injury, and inflammation [38,39].

The effects of alcohol on the liver are even more prominent after bariatric surgery. Around 33% of patients who undergo bariatric surgery develop alcohol use disorder, and up to 5% develop cirrhosis secondary to alcohol-associated liver disease [40]. Patients experience a shorter time to peak blood alcohol concentration and an increased subjective feeling of intoxication compared with presurgical levels. Therefore, the AGA recommends evaluating alcohol use in potential candidates for bariatric surgery due to the significant changes in alcohol metabolism post-surgery [41].

Additionally, there is variability in the way alcohol is processed across different individuals and requires further investigation. Therefore, according to the AASLD guideline recommendations, patients with significant hepatic fibrosis should abstain from alcohol completely, as this will lower the risk of fibrosis progression and hepatic/extrahepatic malignancies [2].

### 5.4. Coffee Consumption

In epidemiological studies and meta-analyses, coffee consumption was demonstrated to reduce the risk of MASLD and liver fibrosis [42,43,44]. A metanalysis was performed to compare coffee consumption and its effect on liver disease. The results show that a coffee intake of 3 cups per day was observed to lower the risk of MASLD compared with <2 cups per day. Beyond 3 cups, the data did not suggest any additional benefits [45]. This protective effect is attributed to caffeine, which is a nonselective adenosine receptor antagonist. Caffeine was shown to inhibit the proliferation of stellate cells, thereby mitigating the risk of liver fibrosis [46]. Other compounds found in coffee, such as cafestol and kahweol, modulate multiple enzymes engaged in the detoxification of carcinogens. Consequently, they hold the potential to reduce the risk of hepatocellular carcinoma [47]. The presence of chlorogenic acids, which is a potent antioxidant in coffee, was shown to attenuate liver fibrogenesis by mitigating oxidative stress [48].

The consumption of regular filtered coffee was found to have a more protective effect on the liver and lowered fibrosis compared with consuming espresso [49]. This may be explained by the light roast of regular coffee, which makes it retain compounds such as chlorogenic acids. Given these multiple benefits, it is recommended by AASLD guidelines to drink at least 3 cups of black caffeinated coffee daily [2].

### 5.5. Exercise

The effects of exercise were shown to reduce the hepatic fat content across the spectrum of MASLD, independent of weight loss. Numerous studies, including a metanalysis of sixteen randomized controlled trials, demonstrated the effects of regular exercise on peripheral insulin sensitivity and hepatic de novo lipogenesis [50,51,52]. A study by O’Gorman et al. examined the impact of a 12-week moderate-to-vigorous aerobic exercise on liver histology in the absence of dietary changes. The results demonstrated significant hepatic histological improvement in hepatic fibrosis and hepatocyte ballooning on repeat liver biopsies [53]. In another RCT that involved 949 diabetic or obese patients, an 8-week exercise program where patients were randomized to a high-intensity interval (HIIT) exercise or moderate-intensity continuous (MICT) aerobic exercise was implemented. The outcomes demonstrated that the patients enrolled in the HIIT programs had reduced intrahepatic triglycerides and improved quality of life compared with the MICT group [54]. In contrast, a study conducted by Eckard et al., which examined a less vigorous combined aerobic–resistance intervention over 6 months, yielded inconclusive results [55]. This disparity illustrates the significance of exercise intensity, implying that a more intensive regimen, such as moderate-to-vigorous aerobic activity, may be necessary to induce histological improvements [56].

Hence, according to the AASLD guidelines, individuals are advised to engage in a minimum of 150 min per week of moderate-intensity physical activity, incorporating both aerobic and resistance exercises [2,57]. Similarly, the American Gastroenterological Association (AGA) recommends 150–300 min of moderate-intensity exercise or 75–150 min of vigorous-intensity exercise weekly for patients with MASLD [22]. Patients must be encouraged to pursue physical activity to the fullest extent possible. Tailored exercise recommendations can contribute to enhancing sustainability and promoting weight loss [2].

## 6. Pharmacological Interventions

### 6.1. Vitamin E

Vitamin E possesses antioxidant properties that are believed to be beneficial for MASLD. It helps to ameliorate oxidative stress, inhibit hepatic de novo lipogenesis, and downregulate proteins involved in lipid synthesis [58].

In the PIVENS trial, 247 patients with MASLD were randomized to pioglitazone 30 mg daily (80 patients) vs. vitamin E 800 IU daily (84 patients) vs. a placebo (83 patients) for a total of 96 weeks. The primary outcome of the study was to investigate the improvement in the histologic features of MASLD. The results indicate that patients on the vitamin E therapy had an improvement in steatohepatitis compared with those on the placebo (43% versus 19%, *p* = 0.001). Specifically, vitamin E improved lobular inflammation, hepatocellular ballooning, and steatosis. However, it did not show a significant effect on fibrosis [59]. Pioglitazone did not meet the primary endpoint for improvement in MASH compared with the placebo (34% vs. 19%, *p* = 0.04). Although no studies have demonstrated a reduction in fibrosis, a retrospective study by Vilar-Gomez et al. showed the association of vitamin E use with lower rates of hepatic decompensation (37% vs. 62%, *p* = 0.04) and higher transplant-free survival [60].

Despite its positive impact on MASLD, there is concern about the potential risks of using high-dose vitamin E. High-dose vitamin E has been associated with an increased risk of hemorrhagic stroke, although further data are required to confirm this observation [61]. Additionally, there is conflicting data regarding the potential increase in prostate cancer among male patients older than 50 years. One randomized controlled trial by Gaziano et al. demonstrated no significant impact of using vitamin E 400 IU every other day on prostate cancer incidence [62]. Conversely, findings from the SELECT trial revealed an increased risk of prostate cancer associated with the administration of 400 IU of vitamin E, with a hazard ratio of 1.17 (99% CI, 1.004–1.36, *p* = 0.008) compared with the placebo group [63].

Therefore, the AASLD suggests that a dosage of vitamin E 800 IU/day may result in improvements in MASLD; however, caution should be taken particularly with high vitamin E doses [2].

### 6.2. Pioglitazone

Thiazolidinediones (TZDs) are peroxisome proliferator-activated receptor-gamma (PPARγ) agonists. PPARγ is a nuclear receptor that regulates the transcription of the genes involved in glucose and lipid metabolism. By activating the PPARγ receptor, TZDs help to increase the insulin sensitivity in adipose tissue, muscle, and liver. Given that insulin resistance is a fundamental pathophysiological pathway in MASLD, TZDs were explored to determine their potential effect on liver function.

In the PIVENS trial, pioglitazone did not meet the primary endpoint of a ≥2-point reduction in NAS without the worsening of fibrosis; however, it did demonstrate a 47% reduction in MASH compared with the placebo. In contrast, another important study by Cusi et al. randomized 101 patients with biopsy-proven MASH and T2DM to pioglitazone (45 mg per day) versus a placebo for 18 months. There was 51% resolution in MASH noted, with improvements in the mean fibrosis score in the TZD group [64]. Another meta-analysis of eight randomized clinical trials evaluated the impact of TZD therapy on histological features of the liver. The results showed that patients treated with TZD had improvements in advanced fibrosis on a liver biopsy, with a resolution of MASH.

These studies highlight the efficacy of TZDs in possibly halting the progression of MASLD [65]. However, the absence of studies that explored the potential effect of TZDs in patients with cirrhosis necessitates further investigation to evaluate any potential anti-fibrotic effect. Despite their potential benefits, the use of TZDs presents certain risks, including weight gain, osteoporosis, a potential exacerbation of heart failure in individuals with preexisting cardiac dysfunction, and a potential increase in cancer risk [66,67]. While TZDs may ameliorate cardiovascular risk factors, alternative medications, such as sodium-glucose cotransporter-2 (SGLT-2) inhibitors and GLP-1 RA, have been favored due to their capacity to induce weight loss alongside reduced cardiovascular mortality.

According to the AASLD recommendations, pioglitazone 30 mg per day may be used in patients with or without T2DM who have a biopsy-proven diagnosis of MASH. However, it should not be used to treat MASLD without evidence of biopsy-proven MASH until further data are available to improve its efficacy and safety. Caution should be taken when managing patient populations, such as those with heart failure or a history of bladder cancer, as it may increase their risk of complications [2].

### 6.3. Glucagon-like Peptide-1 Receptor Agonists

Glucagon-like peptide-1 receptor agonists (GLP-1 RAs) function similarly to the endogenous hormone glucagon-like peptide-1 (GLP-1) by binding to the pancreatic receptor cells and promoting glucose-dependent insulin secretion, leading to lower blood glucose levels [68]. It also decreases glucagon secretion and slows gastric emptying, which curbs the appetite, reduces food intake, and contributes to weight loss [69]. Although GLP-1 receptors are not expressed on the hepatocytes, Kupffer, or stellate cells, GLP-1 RAs offer indirect benefits in liver disease. They help to improve metabolic parameters, such as glycemic/lipid control and weight loss [70]. Additionally, GLP-1 RAs activate the AMP-activated protein kinase, which helps to suppress hepatic lipogenesis, resulting in lower lipid levels [71]. Studies also demonstrated a reduction in adverse cardiovascular events with certain GLP-1 RAs, such as liraglutide, semaglutide, and dulaglutide. This cardioprotective effect is particularly relevant for patients with MASLD, where cardiovascular disease is known to be the leading cause of death. 

In one of the initial trials investigating GLP-1 RAs, 60 patients with obesity, T2DM, and MASLD were randomized to receive exenatide 10 μg twice daily versus insulin boluses. The results revealed a significant improvement in liver enzymes (ALT and GGT) and steatosis after 12 weeks among patients on exenatide over insulin (*p*-value < 0.001 and *p* < 0.01, respectively) [72]. 

In the 48-week LEAN (Liraglutide Safety and Efficacy in Patients with Non-Alcoholic Steatohepatitis) randomized controlled trial, 1.8 mg of Liraglutide once daily was compared with a placebo in 52 patients with MASH, including those with and without diabetes mellitus over a period of 48 weeks. The primary outcome was histologic resolution of steatohepatitis without the worsening of fibrosis. The results demonstrated a 39% resolution of MASH with Liraglutide versus 9% with a placebo (*p* = 0.019) [73]. The patients experienced improvements in hepatocyte ballooning and steatosis, illustrating a reduction in histological damage. Liraglutide also reduced fibrosis progression compared with the placebo group (9% in the liraglutide group, 36% in the placebo group) [73]. Other notable improvements included increased hepatic and adipose insulin sensitivity, which may have been secondary to weight loss [74]. The study suggested that the effect of Liraglutide on liver disease is multifactorial from cumulative weight loss, improved lipid levels, and enhanced glycemic control.

Another study by Newsome et al. compared the daily subcutaneous administration of semaglutide to a placebo in 320 patients with biopsy-confirmed MASH and stages F1–F3 fibrosis. Patients were randomized to semaglutide 0.1 mg, 0.2 mg, or 0.4 mg once daily vs. a placebo. By week 72, there was biopsy-confirmed MASH resolution without worsening of the fibrosis (40% in the 0.1 mg group, 36% in the 0.2 mg group, and 59% in the 0.4 mg group compared with 17% in the placebo group) [74]. However, there was no significant improvement in the fibrosis stage in comparison with the placebo group (43% in the semaglutide 0.4 mg group versus 33% in the placebo group, *p* = 0.48) [74]. Fibrosis worsened in 5% of patients in the semaglutide 0.4 mg group compared with 19% in the placebo group. The lack of statistically significant improvement in the fibrosis stage could have been due to the limited study duration of 72 weeks.

In the recently published STEP-HfpEF DM trial clinical trial, the impact of semaglutide was assessed in 616 patients with obesity, T2DM, and heart failure with preserved ejection fraction. Patients were randomly assigned to receive semaglutide 2.4 mg once weekly for a duration of 52 weeks. The findings revealed that semaglutide resulted in a significantly greater reduction in body weight compared with the placebo group (−9.8% vs. −3.4%, *p* < 0.001), accompanied by improvements in their overall quality of life [75].

Other combination drugs are available, such as tirzepatide, which is a dual GLP-1 and glucose-dependent insulinotropic polypeptide (GIP) agonist. It demonstrated significant benefits in patients with MASLD and MASH. A recent randomized controlled trial investigated the efficacy and safety of tirzepatide in patients with MASH. It involved 190 participants who were randomly assigned to receive tirzepatide once weekly (5 mg, 10 mg, or 15 mg) or a placebo over 52 weeks. The results show that 55% in the 5 mg group had an improvement in fibrosis by at least one stage compared with 51% in the 10 mg tirzepatide group, 51% in the 15 mg tirzepatide group, and 30% in the placebo group. The reported side effects in the tirzepatide groups were mainly gastrointestinal events [76].

Another combination medication is cotadutide, which is a dual GLP-1 and glucagon receptor agonist that was also shown to improve MASLD. A 19-week RCT tested the efficacy of cotadutide (300 μg, 600 μg) or a placebo in 74 patients with biopsy-proven MASH. The results show a dose-dependent improvement in ALT, AST, markers of liver health (FIB-4 markers), and metabolic parameters (insulin resistance, obesity, and lipid profile). These results demonstrate its efficacy in reducing liver fat and improving liver enzymes (AST and ALT), which may be helpful in treating MASH [77].

The potential side effects associated with the use of GLP-1 RA encompass gastrointestinal symptoms, such as nausea, vomiting, diarrhea, and an increased risk of cholecystitis and pancreatitis. Caution should be taken when prescribing this medication to patients with renal impairment or end-stage renal disease [78]. Notably, GLP-1 RA has not yet received approval for the treatment of MASLD due to the absence of completed phase 3 clinical trials establishing its efficacy. While phase 2 trials showed promising improvements in histological features, further research through a phase 3 trial is necessary to obtain FDA approval. Ongoing trials (NCT02970942) are currently underway to further investigate the effect of GLP-1 RA on MASH.

The American Association of Clinical Endocrinologists 2022 Clinical Practice Guidelines for MASLD recommends the initiation of GLP-1 RA for patients with T2DM with biopsy-proven MASH [79]. Based on the current evidence, semaglutide can be started at 0.4 mg once weekly [64] and liraglutide at 1.8 mg once weekly [73]. Tirzepatide could be started at 5 mg, 10 mg, or 15 mg once weekly [76]. The AASLD has also noted the potential benefits of GLP-1 agonist use but recommends further studies to prove its efficacy [2]. Table 2 summarizes the different randomized clinical trials that explored the effect of GLP-1 RA on MASLD.

### 6.4. Sodium-Glucose Co-Transporter 2 (SGLT-2) Inhibitors

SGLT-2 inhibitors work by inhibiting the glucose uptake in the kidney’s proximal tubule, thereby lowering the plasma glucose levels. As a result, they were shown to improve glycemic control in diabetic patients and reduce cardiovascular complications [80]. Moreover, SGLT-2 inhibitors contribute to improved insulin resistance and weight loss, which are both crucial factors in managing MASLD. In a randomized controlled trial that involved MASLD with type 2 diabetes, the use of SGLT-2 inhibitors showed improvement in the liver fibrosis markers (serum 4 collagen 7 s, FIB-4 index, controlled attenuation parameter, and serum ferritin) [81]. Notably, it also led to significant reductions in liver enzymes, including AST, ALT, and GGT. Further support for their efficacy comes from a post hoc analysis of the CANVAS trial, which showed that treatment with canagliflozin is linked to improvements in liver biochemistry and metabolism, potentially benefiting MASLD patients with liver fibrosis, T2DM, and a high cardiovascular risk [82].

Using a Mendelian randomization approach with genetic scores, research showed a negative association between SGLT-2 inhibition and the development of cirrhosis. It also revealed a significantly lower risk of liver-related complications, such as hepatocellular carcinoma, liver transplant, and death in patients on SGLT-2 inhibitors compared with those on DPP-4 inhibitors [83].

The American Association of Clinical Endocrinology (AACE) recommends the consideration of the potential benefits of SGLT2 in patients with type 2 diabetes and MASLD, as it may reduce hepatic steatosis while offering cardiac and renal effects. Additional research is required to fully understand their impact on liver health and histological benefits [64].

### 6.5. Aspirin

Aspirin acts by inhibiting the cyclooxygenase enzymes, which decreases the production of pro-inflammatory prostaglandins [84]. This anti-inflammatory effect inhibits the activation of the hepatic stellate cells, thus inhibiting the progression of hepatic fibrosis. Aspirin was also shown to upregulate the PPAR-AMPK-PGC-1 pathway, thus regulating the lipid biosynthesis and inflammation [85].

A randomized controlled trial explored the effect of aspirin on MASLD. It involved 80 patients with MASLD who did not have cirrhosis. Patients were then randomized to receive aspirin 81 mg once daily or a placebo for a total duration of 6 months. By the end of the study, the average fat content by magnetic resonance spectroscopy (MRS) was decreased by 6.6% in the aspirin group compared with 3.6% in the placebo group [86]. However, this study was limited by its small sample size and lack of long-term follow-up. The study primarily addressed hepatic steatosis, and it did not explore outcomes related to cirrhosis progression or mortality. Furthermore, it is crucial to evaluate the risk–benefit profile in MASLD patients who do not meet the criteria for routine aspirin use [86].

Another meta-analysis analyzed aspirin use in 1856 patients with liver disease or had strong risk factors of liver disease (such as viral hepatitis, alcohol use, and MASH). The results show a significantly lower composite liver fibrosis index (0.24 standard deviation units lower; 95% CI 0.42 to 0.06, *p* = 0.009) in patients who used aspirin [87]. It was also shown to reduce the risk of HCC and liver-related death without significant risk of bleeding [88]. Considering these findings, low-dose aspirin (81 mg) emerged as a potential therapeutic option for MASLD; however, further studies with a larger sample size are recommended to validate these results and ascertain their benefits on liver disease.

### 6.6. Pemfibrate

Pemafibrate is a highly selective peroxisome proliferator-activated receptor (PPAR)-α modulator that is found to be more potent than other PPAR-α modulators [89]. By upregulating the expression of uncoupling protein 3 in the liver, it enhances triglyceride hydrolysis and fatty acid β-oxidation, consequently reducing hepatic steatosis [89,90].

In a prospective study involving patients with MASLD and dyslipidemia, a 48-week treatment with Pemafibrate led to reductions in liver enzymes, including ALT, AST, and alkaline phosphatase [91]. Similarly, an analysis of patients on Pemafibrate for at least one year revealed improvement in the hepatic steatosis index (HIS) and FIB 4 index, though no significant changes in the fibrosis score (NFS) were reported [92]. Another study compared the effects of combining Pemafibrate with a mild low-carbohydrate diet, revealing improvement in liver stiffness through magnetic resonance elastography irrespective of weight changes [93].

These findings indicate the potential benefit of Pemafibrate in improving liver enzymes and fibrosis, suggesting its therapeutic benefit for MASLD. Further studies are required to establish its long-term safety and efficacy.

### 6.7. Resmetirom

Resmetirom, which is a thyroid hormone receptor-β (THR-β) selective agonist, has been developed for the treatment of MASLD. Its activation of THR-β is associated with a cascade of metabolic effects, such as lowering lipids, increasing bile synthesis, and promoting fat oxidation [94]. Additionally, Resmetirom suppresses STAT3 and NF-κB, which are key pathways that contribute to inflammation and fibrosis in MASLD [95].

In the MAESTRO-NAFLD-1 trial, Resmetirom’s safety and efficacy were assessed in patients with MASLD and MASH. A total of 1400 patients were randomized to 80 mg of Resmetriom, 100 mg of Resmetirom, or a placebo. After 52 weeks, significant reductions in hepatic fat (28.8% for 80 mg and 33.9% for 100 mg) compared with a placebo were observed, along with reductions in LDL-C, apolipoprotein B, and triglycerides [96].

The MAESTRO-NASH trial explored the effect of daily Resmetirom in adults with biopsy-confirmed MASH and liver fibrosis (ranging from F0 to F4) [97]. In this study, 966 patients were also randomized to Resmetirom (80 mg or 100 mg) or a placebo. At 52 weeks, Resmetirom recipients had MASH resolution without the worsening of fibrosis compared with the placebo group (25% in the 80 mg group, 29.9% in the 100 mg group, and 9.7% in the placebo group). Additionally, the study demonstrated improvement in liver fibrosis by at least one stage without worsening the nonalcoholic fatty liver disease activity score [98].

The ongoing MAESTRO-NASH-OUTCOMES trial is enrolling patients with well-compensated MASH to explore Resmetirom’s potential to slow the progression of hepatic decompensation [97].

In all the published trials, Resmetirom demonstrated no significant impact on body weight, glucose levels, or insulin resistance. However, it exhibited a significant impact in reducing the LDL cholesterol, triglycerides, apolipoprotein B, and lipoprotein(a), thereby potentially lowering cardiovascular complications [97]. Compared with the placebo group, patients that received Resmetirom experienced gastrointestinal symptoms, such as diarrhea, nausea, vomiting, or constipation. The highest incidence of these adverse events was noted among individuals that received the 100 mg/day dosage [97]. These symptoms manifested early in the treatment regimen, typically within the initial 12 weeks. By week 52, trial discontinuations were more common in the 100 mg Resmetirom group compared with the other dosage groups, which was largely attributed to adverse gastrointestinal events (7% in the 100 mg group, 2% in the 80 mg group, and 2% in the placebo group). The occurrence of serious adverse events, encompassing gastrointestinal, cardiovascular, respiratory, nervous system, and musculoskeletal disorders, was comparable across all trial groups (ranging from 10.9% to 12.7%) [97]. Resmetirom was also associated with elevated levels of plasma sex hormone-binding globulin (SHBG), but it did not affect thyroxine-binding globulin [97]. Consequently, the long-term implications of elevated plasma SHBG levels remain uncertain and may potentially lead to clinically significant changes in the gonadal axis [97].

Overall, given its promising results in reducing hepatic fat content, circulating lipids, and cardiovascular events in MASLD, Resmetirom recently gained conditional approval by the Food and Drug Administration (FDA) for the treatment of non-cirrhotic MASH with moderate-to-advanced fibrosis [57]. The recommended dose is 80 mg to 100 mg once daily.

Obtaining extended-term data would greatly enhance our comprehension of the medication and its long-term effects on MASLD. Furthermore, since the studies only involved patients over 18 years old, we lack information regarding the safety and effectiveness of its use in individuals under 18. It is imperative to implement regular monitoring for potential risks associated with gonadal and bone diseases. Additionally, when Resmetirom is co-administered with certain medications, like statins, clopidogrel, or cyclosporine, considering dose adjustments, such as reducing it to 60 mg, can help to mitigate potential drug–drug interactions [99].

### 6.8. Statins

A post hoc analysis of the Greek Atorvastatin and Coronary Heart Disease Evaluation (GREACE) trial focused on patients with coronary heart disease and abnormal liver tests that are possibly indicative of MASLD. The results show that among the 437 patients with moderately abnormal liver tests, those who received atorvastatin 24 mg/day (227) had substantial improvement in liver tests compared with the placebo group (210). Additionally, the cardiovascular benefit was significant, as it reduced cardiovascular events [100].

In the Initiating Dialysis Early and Late (IDEAL) trial, the effect of high-dose atorvastatin (80 mg/day) versus usual-dose simvastatin (20 mg/day) was investigated in patients with myocardial infarction. In a post hoc analysis, it was notable that the use of high-dose atorvastatin lowered LDL-C levels, reduced cardiovascular events, and reduced elevation in ALT compared with moderate-intensity statin [101]. Although high-dose atorvastatin raises concerns about potential hepatotoxicity, the trial did not report significant liver-related adverse events, suggesting the safety of using high-dose statin. Therefore, the AASLD guidelines recommend the use of lipid-lowering therapy in patients with MASLD and dyslipidemia [57]. It should be avoided in patients with decompensated liver disease [57].

### 6.9. Metformin

Since insulin resistance is one of the key drivers in the pathophysiology of MASLD, the use of metformin was investigated to determine whether it is beneficial for MASLD management. A meta-analysis by Li et al. included 9 studies with 417 participants over a time period of 4 to 12 months. It compared metformin with a placebo in the treatment of MASLD. The results show improvement in ALT, AST, and BMI, but not in histological changes, such as steatosis, inflammation, hepatocellular ballooning, or fibrosis [102]. Further, larger randomized controlled trials should be conducted to fully understand the effect of metformin on liver disease. The AASLD guidelines do not recommend the use of metformin for treating MASLD/MASH.

### 6.10. Anti-Obesity Medications

Various FDA-approved anti-obesity medications are now available for individuals with a BMI of 27 kg/m^2^ or greater with at least one weight-related comorbidity or for those with a BMI of 30 kg/m^2^ or greater. Previously, we explored GLP-1 RAs, both of which have FDA approval for effective weight management. Beyond GLP-1 RAs, there exist other pharmacological options, including Phentermine, Naltrexone/Bupropion, Orlistat, and Phentermine/Topiramate [103].

Phentermine, which is a potent appetite suppressant, is typically prescribed for short-term use to curb food intake. Meanwhile, the synergistic combination of naltrexone/bupropion modulates brain chemistry to diminish food cravings while enhancing feelings of fullness. Orlistat operates by inhibiting the absorption of dietary fat within the intestines. Lastly, the Phentermine/Topiramate pairing exerts its effects by reducing appetite and amplifying sensations of satiety [103].

Studies showed that Orlistat helps to reduce liver fat content and improve the histologic features of MASH [104]. However, the existing literature offers limited insight into the utilization of anti-obesity medications for MASLD, necessitating further research to understand their impact on liver-related pathologies.

### 6.11. Other Therapeutic Options

There are other medications that are being explored for the treatment of MASLD. The farnesoid X nuclear receptor (FXR) ligand obeticholic acid showed promising results in reducing liver fibrosis, as shown in the FLINT [105] and REGENERATE [105] trials; however, there was an unfavorable effect on the lipid profile. Further studies are required to determine its pathophysiology, optimal dosing, and side effects. Other agents, including fibroblast-growth-factor 19, fibroblast-growth-factor 21, and novel lipase synthesis inhibitors, are currently being investigated and results are eagerly awaited. Table 3 summarizes the available MASLD treatments with their clinical benefits and guideline recommendations.

## 7. Surgical Interventions

### 7.1. Bariatric Surgery

Bariatric surgery is recommended for individuals with a BMI ≥ 35 kg/m^2^, regardless of co-morbidities; T2DM and a BMI ≥ 30 kg/m^2^; and a BMI of 30–34.9 kg/m^2^ who do not achieve substantial or durable weight loss or co-morbidity improvement using nonsurgical methods [106]. Bariatric surgery was shown to improve hepatic fibrosis, resolve MASH, induce sustained weight loss, improve diabetes, and reduce all causes of morbidity and mortality [107,108,109].

The effects of bariatric surgery were explored in the SPLENDOR study that analyzed 1158 patients with biopsy-proven fibrotic MASH without cirrhosis managed with bariatric surgery compared with the nonsurgical approach. Patients who underwent bariatric surgery had a 12.4% reduction in major adverse liver outcomes and a lower risk of major cardiovascular events [109]. A systematic review by Bower et al. investigated the effects of bariatric surgery in obese patients with MASLD. The study compared the preoperative to post-operative liver enzymes and histological liver changes. The results demonstrated histological improvements, including reductions in steatosis by 50.2% (95% CI: 35.5–65.0), fibrosis by 11.9% (95% CI: 7.4–16.3%), hepatocyte ballooning by 67.7% (95% CI: 56.9–78.5), and lobular inflammation by 50.7% (95% CI: 26.6–74.8%). Additionally, there were improvements in the liver enzyme levels of ALT, AST, and ALP [110]. Another study investigated the effects of bariatric surgery on patients with MASH over 5 years. It included 180 patients with biopsy-proven MASH. Liver biopsies were obtained at the time of bariatric surgery, followed by repeat biopsies 1 and 5 years later. The results at 5 years with repeat biopsies show 84% improvement in MASH without worsening of the fibrosis. Fibrosis decreased by one stage in 70.2% of patients, completely disappeared in 56% of all patients, and 45.5% in patients with baseline bridging fibrosis. There was no significant recurrence in MASH between 1 and 5 years [111].

In cases with inadequate weight loss following bariatric surgery, patients may experience persistent MASH. Due to the exclusion of individuals with advanced hepatic fibrosis and cirrhosis during presurgical screening, limited information is available regarding the effect of surgery in these populations. Additionally, patients with advanced cirrhosis are at higher risk of surgical complications, including hepatic decompensation and postoperative mortality [112,113]. In patients with decompensated cirrhosis, bariatric surgery may be considered in certain circumstances, often in conjunction with a liver transplant.

According to the current AASLD guidelines, bariatric surgery is recommended for patients who meet the criteria for metabolic weight loss surgery. Various types of bariatric procedures are available, such as Roux-en-Y bypass, sleeve gastrectomy, adjustable gastric banding, and biliopancreatic diversion. However, there are no definitive recommendations regarding the choice of bariatric surgery type. Instead, a multidisciplinary approach is necessary to assess the benefit–risk profile of each procedure for individual patients [64].

### 7.2. Intragastric Balloons

Intragastric balloons (IGBs) are also utilized as a surgical intervention that is used to induce weight loss. A meta-analysis was conducted to review and analyze the available literature on the effect of intragastric balloons on liver enzymes and MASLD markers [113]. The study assessed liver enzymes, including ALT and GGT, as well as MASLD markers (based on imaging and biopsy) before the insertion of the intragastric balloon and after its removal at 6 months. The results show reductions in ALT by −10.02 UL (95% CI, −13.2, −6.8), GGT by −9.82 U/L (95% CI, −12.9, −6.8), and BMI by −4.98 kg/m^2^ (−5.6, −4.4) with IGB therapy. Hepatic steatosis improved after 6 months according to MRI imaging (fat fraction: 16.7 ± 10.9% to 7.6 ± 9.8%, *p* = 0.003) and ultrasound (severe liver steatosis: 52% to 4%, *p* < 0.0001). The histological NAFLD activity score was also reduced compared with the control group [113]. Another meta-analysis by Chandan et al. explored nine different studies that included 442 intragastric balloon placements in patients with MASLD. The results illustrate a 79.2% histological and imaging improvement in steatosis. Additionally, the Homeostatic Model Assessment of Insulin Resistance (HOMA-IR) was improved in 64.5% of the patients. The nonalcoholic activity score improved in 83.5% of patients with a 93.9% reduction of liver volume evident according to CT scans [112]. However, according to the American Association of Clinical Endocrinology (AACE) and AASLD, endoscopic bariatric procedures (including IGB insertion) should not be recommended in patients with MASLD due to the limited evidence available and the concerns about the generalizability of the trial results. Higher quality research is recommended to explore the IGB efficacy and safety profile in patients with MASLD.

## 8. Conclusions

In summary, the approach to managing MASLD is multifaceted, with a primary focus on weight loss and addressing underlying comorbidities. Weight loss remains the most impactful treatment strategy, which can be achieved through a spectrum of interventions. Lifestyle modifications, such as adopting healthy dietary habits, like the Mediterranean diet, and increasing physical activity were shown to promote weight loss and improve comorbidities in MASLD. Other therapeutic options, including medications and surgical interventions, are also available. The early identification and effective management of MASLD are crucial in preventing disease progression to liver fibrosis and cirrhosis. Ongoing research into emerging pharmacological therapies is currently under investigation and holds promise for uncovering additional benefits in treating MASLD.

## Figures and Tables

**Table 1:** NAFLD activity score and fibrosis score. This table describes the scoring and staging requirements for the NAFLD activity score and fibrosis score.

NAFLD Activity Score (NAS)				
**Score**	**0**	**1**	**2**	**3**
Steatosis	<5%	5–33%	>33–66%	>60%
Lobular inflammation	None	<2 foci/20× optical field	2–4 foci/20× optical field	>4 foci/20× opticalfield
Hepatocyte ballooning	None	Few	Many	
**Fibrosis Score**				
**Stage**	**1**	**2**	**3**	**4**
Histological findings	Mild–moderate pericellular fibrosis	Pericellular and portal fibrosis	Bridging fibrosis	Cirrhosis

**Table 2:** Summary of GLP-1 receptor agonists and GLP-1 and glucagon receptor agonist randomized clinical trials.

Clinical Trial	Medication	Patient Population	Study Design	Results
**Armstrong et al.** **[73]**	Liraglutide 1.8 mg daily	52 patients diagnosed with MASH +/− DM	Randomized to liraglutide 1.8 mg daily vs. placebo for 48 weeks	Liraglutide group had improved hepatocyte ballooning, steatosis, reduced fibrosis progression, and increased insulin sensitivity
**Newsome et al.** **[74]**	Semaglutide 0.1, 0.2, or 0.4 mg once daily	320 patients with MASH and liver fibrosis at stage F1, F2, or F3	Randomized to semaglutide 0.1, 0.2, or 0.4 mg once daily vs. placebo for 72 weeks	Semaglutide improved MASH with no difference in fibrosis.
**Kosiborod et al.** **[75]**	Semaglutide 2.4 mg weekly	616 patients with heart failure with preserved ejection fraction, BMI > 30, and T2DM	Randomized to semaglutide 2.4 mg weekly vs. placebo for 52 weeks	Semaglutide group had a reduction in body weight (by 9.8%) compared with a placebo (3.4%)
**Loomba et al.** **[76]**	Tirzepatide 5 mg, 10 mg, or 15 mg once daily	190 patients with biopsy-proven MASH	Randomized to tirzepatide 5 mg, 10 mg, or 15 mg vs. placebo for 52 weeks	Tirzepatide improved fibrosis by at least one stage
**Shankar et al. [77]**	Cotadutide 300 μg or 600 μg	74 patients with biopsy proven MASH	Randomized to cotadutide 300 μg or 600 μg vs. placebo for 19 weeks	Cotadutide improved ALT, AST and liver fibrosis markers (reduced FIB-4 scores)

**Table 3:** Summary of different medications available, their indications, and potential side effects.

Medication	FDA-Approved Indication	Clinical Benefits		Potential Side Effects	Guideline Statements
		Liver-Related	Non-Liver-Related		
Vitamin E	N/A	Improves steatosis but has no proven benefit on fibrosis	N/A	Hemorrhagic stroke Increased risk of prostate cancer	It can be considered in select individuals without diabetes as it improves NASH in some patients.
Pioglitazone	T2DM	Improves steatosis and MASH	CV risk reduction Improves insulin sensitivity	Weight gain Heart failure exacerbation Bone loss	It can be considered for patients with T2DM, as it improves MASH [2].
GLP-1RA *Liraglutide* *Semaglutide* *Tirzepatide*	T2DM Obesity	Improves steatosis but has no proven benefit on fibrosis	CV risk reduction Improves insulin sensitivity Weight loss	Gastrointestinal side effects (gallstones, pancreatitis)	It can be considered for its approved indications (T2DM/obesity) in patients with NASH, as it confers a cardiovascular benefit and improves NASH.
SGLT-2 inhibitors	T2DM	Improves steatosis	Improves CV and renal outcomes Helpful for heart failure Improves insulin sensitivity Modest weight loss	Genitourinary infections Volume depletion Bone loss	The therapeutic impact of SGLT-2i on liver histology needs to be better defined.
Aspirin	CV * indications	Improves steatosis and potentially improves fibrosis	CV risk reduction	Potentially increased risk of bleeding	N/A
Pemafibrate	Hypertriglyercidemia	Improves steatosis and liver enzyme markers	Favorable effect on lipid profile Improves insulin sensitivity	Gastrointestinal symptoms Myalgia	N/A
Resemtirom	MASLD	Improves steatosis, MASH, and fibrosis	Improves CV outcomes	Gastrointestinal side effects Increased SHBG **	Approved by FDA for MASLD treatment.

* CV: cardiovascular ** SHBG: sex-hormone binding globulin.
